# Probiotics in Traumatic Brain Injury: New Insights into Mechanisms and Future Perspectives

**DOI:** 10.3390/jcm13154546

**Published:** 2024-08-03

**Authors:** Diamantoula Pagkou, Evangelos Kogias, Nikolaos Foroglou, Katerina Kotzampassi

**Affiliations:** 1 Department of Neurosurgery, AHEPA University Hospital, Aristotle University of Thessaloniki, Kiriakidi 1, 54636 Thessaloniki, Greece; kogias@auth.gr (E.K.); nforoglou@auth.gr (N.F.); 2 Department of Surgery, Aristotle University of Thessaloniki, Kiriakidi 1, 54636 Thessaloniki, Greece; kakothe@yahoo.com

**Keywords:** gut dysbiosis, probiotics, gut–brain axis, gut microbiome, traumatic brain injury (TBI)

## Abstract

Traumatic brain injury (TBI) is a serious global public health issue, recognized as a chronic and progressive disease that can affect multiple organs, including the gastrointestinal (GI) tract. Research shows that there is a specific link between the GI tract and the central nervous system, termed the gut–brain axis, which consists of bidirectional exchange between these two. Several preclinical and clinical studies have demonstrated intestinal barrier dysfunction, intestinal inflammation and gut dysbiosis in patients with TBI. It is proven that probiotics can modulate the inflammatory process and modify gut microbiota. Numerous animal studies and human clinical trials have proven the effectiveness of selected bacterial strains as an adjuvant treatment in reducing inflammation, infection rates and time spent in intensive care of hospitalized patients suffering from brain injury. Thus, this review summarizes the current evidence regarding the beneficial effects of probiotic administration in patients suffering from TBI-related complications. This review will help identify novel therapeutic strategies in the future as probiotics have an extensive history of apparently safe use.

## 1. Introduction

Traumatic brain injury (TBI) occurs when an external mechanical force, most commonly after traffic accident, fall, gun shot or sport injury, acutely disrupts the normal function of the brain [1,2]. TBI represents an important public health issue due to its contribution to transient or permanent disability, decreased lifespan and trauma-related death. The global incidence of all-severity, all-cause TBI is estimated at 939 cases per 100.000 people; thus. 69 million people suffer a TBI every year worldwide [3]. The incidence of TBI globally is rising, mainly owing to injuries associated with the increased use of motor vehicles, particularly in middle-income and low-income countries [4]. Moreover, the recent literature shows that more than 1.1% of the US population lives with TBI, and more than 40% of those with moderate-to-severe injuries have long-term disabilities with healthcare cost estimates ranging from USD 56 to USD 221 billion annually [5].

Patients with severe TBI usually undergo prehospital intubation and the majority of them require tracheostomy during intensive care unit (ICU) stays. These patients often become dependent on mechanical ventilation that increases the ICU length of stay and the risk of severe complications such as mechanical-ventilation-associated pneumonia as well as respiratory and renal failure and sepsis [6,7]. Moreover, recent data strongly suggest that stress-induced hyperglycemia is common among patients with moderate–severe TBI and is associated with high mortality rates [8,9,10]. Additionally, brain-injured individuals in the ICU are subjected to nutritional changes and long-term antibiotic therapy; thus, they have a greater risk of suffering from gastrointestinal (GI) symptoms such as enteral feeding intolerance, gut dysmotility, diarrhea and reflux. Patients suffering from TBI are more likely to develop dysbiosis, a pathological imbalance of gut microbial species and reduction in gut biodiversity [11,12].

The human gut microbiota (GM) refers to a vast number of microorganisms, such as bacteria, fungi, archaea and viruses, which colonize the gastrointestinal tract (GI), and interact with one another and with the human host [13]. On the other hand, the gut microbiome is the term given to describe the collective genomes of these microorganisms [14]. GM mainly includes six phyla, namely, Bacteroidetes, Firmicutes, Proteobacteria, Verrucomicrobia, Actinobacteria, and Fusobacteria [15]. The composition of the GM is crucial for maintaining health and has been linked with vital functions such as protection against pathogens, immune regulation, metabolism and vitamin production. There is growing evidence that changes in the GM’s composition, called dysbiosis, are correlated with neurological and neurocognitive disorders [16].

The relationship between the GM and the brain has been a subject of numerous studies for decades. The bidirectional linkage between the GM and the central nervous system (CNS), termed the “gut microbiome–brain axis” (GMBA), facilitates gut motility, intestinal mucosal integrity, and immunologic homeostasis. TBI results in disruption of the GM starting within hours following injury and is associated with chronic gut dysfunction [17]. Taking into consideration that gut dysbiosis in TBI patients is one of the leading causes of organ failure and multiple system complications, targeting the GM and remodeling the balance of gut microbial community should be considered an important therapeutic goal during hospitalization [18]. In the last decade, many therapeutic strategies focused on restoring the GM have been designed. These include early enteral nutrition, administration of probiotics, appropriate use of antibiotics and fecal microbiota transplantation [19,20].

Recent research supports the option that probiotic administration beginning early after trauma could beneficially affect the GM through the maintenance of intestinal barrier integrity and immunomodulation activity, resulting in lower TBI-related complication rates [21,22]. Several animal and human studies focused on alterations in the GM’s composition following TBI demonstrated the potential role of probiotic consumption in attenuating gut dysbiosis [23]. The purpose of this review is to summarize the current evidence supporting the beneficial effects of probiotic administration in preclinical and clinical studies of TBI-induced GM dysbiosis, and whether the modification of GM could influence the clinical outcome of TBI.

## 2. The Gut Microbiota–Brain Axis (GΜBA)

In recent years, there has been a growing interest in the bidirectional communication between the brain and GM [24]. The GMBA refers to a complex network, linking the central nervous system and the GM, modulated by neurons, neurotransmitters, immune system molecules and hormones. Numerous studies have shown the influence of the GM on cognitive capabilities such as learning and memory, psychiatric and neurodevelopmental disorders and neurodegenerative diseases [25].

The top–down pathway between the brain and GM involves the autonomic and the enteric nervous system, as well as the hypothalamic–pituitary–adrenal (HPA) axis. The autonomic and the enteric nervous system regulate important gut functions such as intestinal homeostasis, motility and secretion of gastric acid and bile, intestinal barrier permeability, and mucosal immune response, thus leading to microbial diversity [26]. Stress-induced HPA axis activation leads to the production of stress hormones such as catecholamines and glucocorticoids, which increase intestinal permeability and alter the GM’s composition, resulting in bacterial inflammatory cytokine production, including interleukin-1 (IL-1), interleukin-6 (IL-6) and tumor necrosis factor-α (TNF-α) [27].

Recent studies have provided insights into the possible pathways that connect the GM to the brain. In fact, recent evidence widely suggests that the GM modulates brain development, function and behavior [28]. The GM affects the host through neural, immune, neuroendocrine and metabolic pathways. The key communication routes between the enteric microbiota and the brain are the vagal nerve, tryptophan metabolites, and microbial metabolite products, such as short-chain fatty acids (SCFAs) and lipopolysaccharide (LPS) [29]. SCFAs are among the most important class of gut microbiota bio-products. SCFAs are produced in the colon mainly by bacterial fermentation of dietary fibers and resistant starch. Butyrate, acetate and propionate are the most abundant SCFAs in the human body. SCFAs improve gut health through a number of local effects, ranging from the maintenance of intestinal barrier integrity to mucus production, and protection against inflammation. Furthermore, SCFAs are able to cross the blood–brain barrier and have been shown to regulate microglia homoeostasis, which is required for proper brain development and brain tissue homoeostasis [30]. Another decisive bacterial metabolite is LPS, which is principally derived from the cell walls of Gram-negative enterobacteria. LPS can enter into the systemic circulation via intestinal epithelial tight junction’s defects and cross the BBB, causing neuroinflammation [31]. Moreover, microbial-induced metabolism of dietary tryptophan, particularly by certain species of the Lactobacillus genus, results in the production of neuroprotective and neurotoxic metabolites [32]. Nowadays, it is clear that certain bacteria are strain-specifically able to produce different essential neurotransmitters and specific neuromodulators. Neurotransmitters, such as gamma-aminobutyric acid (GABA), serotonin, catecholamines and acetylcholine, are produced by bacteria belonging to *Lactobacillus*, *Bifidobacteria*, *Enterococcus*, and *Streptococcus* species. These neurotransmitters induce epithelial cells to release molecules that, in turn, have the ability to modulate neural signaling within the enteric nervous system and subsequently control brain function and behavior. In the GI epithelium, enterochromaffin cells are responsible for producing 90% of the body’s serotonin [33].

## 3. Effects of TBI on Gut Microbiome

TBI consist of two phases: the primary injury and the secondary one. The primary injury takes place within seconds to minutes following trauma and involves direct damage to neurons as a result of the initial local impact. It can result in intracranial hemorrhage, brain edema, acute brain ischemia and, in severe cases, diffuse axonal injury. The secondary injury follows after hours or weeks in response to the primary event, and induces a complex cascade of ischemic, inflammatory, immunological and cytotoxic processes, leading to blood–brain barrier disruption, and thus to secondary brain damage [34].

TBI is increasingly recognized not only as an acute condition but also as a chronic disease with long-term devastating sequelae outside the nervous system. Gut dysbiosis, intestinal barrier dysfunction and chronic enteric inflammation are parallel changes that happen after TBI, which could ulteriorly aggravate the injury profile. Dysbiosis is related to the imbalance between bacteria that normally inhabit the intestine and virulent bacteria, resulting in beneficial bacteria decrease, pathogenic bacteria overgrowth and overall bacterial diversity loss. The phenomenon of dysbiosis could absolutely cause misinterpretation of gut signals in the brain and alter the everyday physiological functions between gut and CNS [31]. Although dysbiosis has been thoroughly studied in the pathogenesis of GI neurodegenerative and metabolic disorders, the investigation of TBI-induced gut dysbiosis in preclinical and clinical models remains very limited and only recently has it been explored [35].

Intestinal barrier dysfunction is often considered a major outcome among TBI patients that further contributes to long-term complications [36,37]. Experimental TBI models in mice demonstrated decreased villous length, crypt depth, and surface area, epithelial cell death, and loss of tight-junction proteins such as zonulin-1 (ZO-1) and occludin creating a “leaky gut” [38,39]. In a *Drosophila melanogaster* model of closed-head brain injury, disruption of the intestinal barrier and GI permeability was observed, mediated by adrenergic signaling [37]. Damaged intestinal epithelium leads to severe consequences such as malnutrition, electrolyte disorders, bacterial translocation, systemic inflammatory response, sepsis, and multiple organ failure, which further increase mortality and prolongs the length of hospital stay. Common GI symptoms following TBI include feeding intolerance, dyspepsia and chronic diarrhea, causing additional morbidity [40].

According to numerous studies on TBI, there is an increasing evidence that acute and chronic inflammatory events contribute to secondary damage and long-term neuronal disorders. The neuroinflammatory response following TBI is the result of complex interactions between components from both the CNS and periphery and has been shown to be involved in sepsis, systemic inflammatory response syndrome (SIRS) and, consequently, multiple organ failure syndrome [41]. In the gut, TBI induces an increased enteric glial cells activation as an adaptation to chronic brain-derived stimulus mechanism, serving to promote mucosal homeostasis and attenuate the inflammation in response to intestinal barrier disruption [42,43,44]. Attenuating the detrimental aspects of the inflammatory response is a promising strategy to ameliorate the secondary injury.

### 3.1. Effects of TBI on the Gut Microbiome in Animal Studies

Animal studies have shed light on the potential interaction mechanisms between microbiota and their host organisms. In recent decades, numerous animal experimental models have been developed to mimic various aspects of human TBI. Mice and rats are the most commonly used animal models for studying TBI. Controlled cortical impact injury, fluid percussion injury, weight-drop TBI, and repeated mild TBI models are among the most used in animal TBI experimental studies [45]. The controlled cortical impact model uses an electromagnetic device to produce focal brain contusion injury. The fluid percussion injury model creates a rapid fluid pulse injection, through a craniotomy, directly to the intact dural surface, creating a diffuse deformation of the brain. In the weight-drop model, brain injury is caused by a free-failing weight which directly impacts the animal exposed brain [46].

Houlden et al. [47] identified a positive correlation between gut dysbiosis and injury severity in a TBI mice model 72 h after a closed-head impact. This study demonstrated a significant decrease in the levels of *Prevotellaceace* (Firmicutes phylum) and an increase in the levels of *Peptococcaceae* (Bacteroidetes), correlated with the extent of the injury. In another study, Waligora-Dupriet et al. [48] found significant differences in GM after head injury induction in 12 male rats in comparison to controls: a reduced abundance of the beneficial anaerobic groups within the Bacteroidetes, Actinobacteria and Firmicutes phyla and increases in facultative aerobes of the Enterobacteriaceae family. Treangen et al. [49] demonstrated in mice a rapid significant decrease in *Lactobacillus gasseri*, *Ruminococcus flavefaciens*, and *Eubacterium ventriosum* and a significant increase in *Eubacterium sulci*, and *Marvinbryantia formatexigens* as early as 24 h post-TBI.

In a controlled cortical impact rodent model of moderate TBI in rodents, Nicholson et al. [50] observed changes in the gut microbiota that favor the pathogenic Gram-negative species of *Bacteroidetes* and *Proteobacteria* over the beneficial from the *Firmicutes* phylum as early as 2 h post-injury. These alterations remained for more than a week, causing an increase in gut permeability. The same finding of long duration of GM disruption was also documented in a recent study, where fecal samples were studied both at the 7th and the 28th post-TBI days. It is of interest to note that authors referred to a decrease in the population of *Akkermansia municiphila*, a beneficial bacterium belonging to *Verrucomicrobia* [51,52]. However, in a similar experimental mice TBI model, increased levels of *Akkermansia municiphila* were detected at 1, 2, and 3 days after brain injury, indicating that alterations in the abundance of *Akkermansia municiphila* may be divided into two phases [53].

Finally, a significant decrease in beneficial butyrate producing bacteria—*Agathobacter*, *Faecalibacterium*, and *Eubacterium*—and an increase in species belonging to *Prevotellaceae* and *Rikenellaceae* families were also found more recently by others [54], while a systematic review performed in 2020 also concluded that there was an increase in *Proteobacteria* and *Firmicutes* bacterial populations in all six studies assessed [55].

### 3.2. Effects of TBI on the Gut Microbiome in Human Studies

To date, only four clinical studies have addressed the issue of the potential impact of brain injury on the GM. In 101 patients with moderate-to-severe TBI, feces received by means of rectal swabs revealed colonization by the Proteobacteria phylum, with Enterobacteriaceae forming the largest group [56].

Moderate to severe TBI patients were recently examined by Pyles et al. [57]. The researchers collected fecal samples at the time of the injury as well as 5 years post-TBI. Interestingly, significant alterations in the fecal microbiota were detected 5 years after brain injury. Corynebacterium, Alistipes and Pseudomonas genus were estimated with higher concentrations compared to control profiles.

Brenner et al. [58], in an effort to document the longevity of GM changes, analyzed fecal samples from veterans with a history of brain trauma 28 years before, but no difference was found in alpha and beta diversity between those having experienced TBI of different severities in relation to the control group, namely, veterans without TBI history. On the contrary, Urban et al. [59] found a significant reduction in the Prevotella and Bacteroides species and an increase in the Ruminococcaceae family in permanent care residents who suffered from chronic brain trauma consequences in relation to healthy controls, although there is no information regarding whether they were also residents of the same facilities or not.

## 4. Probiotics and Their Effects on TBI-Related Gut Dysbiosis and Inflammation

According to the Food and Agriculture Organization of the United Nations and the World Health Organization (FAO/WHO), probiotics are defined as “live microorganisms which, when administered in adequate amounts, confer a health benefit to the host [60,61]. Probiotics are currently in the spotlight of research given the growing evidence with regard to their potential effects to prevent and/or restore gut microbial dysbiosis [62,63,64,65], thus improving patients outcomes [66]. It has been demonstrated that TBI patients are at high risk of surgical and nosocomial infections resulting from prolonged hospitalization [67]. A post hoc analysis by Tzikos et al. in multiple-trauma patients with concomitant head injury showed that a probiotic mixture significantly reduced the incidence of surgical site infection [68]. Since growing evidence suggests the ability of probiotics to manipulate the GM, we explore the potential role of probiotics as an effective therapeutic strategy to ameliorate TBI-induced pathology and symptoms. The potential role of probiotics in the treatment of TBI systemic effects has been investigated in several experimental and clinical studies [69].

### 4.1. Animal Studies

In preclinical TBI models, the impact of probiotics on restoring GM and modifying immune system and inflammatory responses is well established. These models demonstrated the efficacy of the administration of several different species of probiotic bacteria, including lactobacilli and butyrate-producing gut bacteria on animal head injury models.

In recent decades, numerous animal experimental models in mice and rats have been developed as an approximation of the actual pathophysiological events of human TBI [70]. Controlled cortical impact injury, fluid percussion injury, weight-drop TBI, and repeated mild TBI models are among the most commonly used in animal TBI experimental studies [45]. The controlled cortical impact injury model uses an electromagnetic device to produce focal brain contusion. In the weight-drop model, brain injury is caused by a free-falling weight which directly impacts the exposed brain of the animal. The fluid percussion injury model takes advantage of a rapid fluid pulse injection, through a craniotomy, directly to the intact dural surface, causing a diffuse deformation of the brain [46].

Li et al. [71] evaluated the effect of *Clostridium butyricum* in a mouse model of TBI induced by weight-drop impact head injury. The mice were intragastrically given *C*. *butyricum* (109 CFU/mL) or placebo once daily for 14 consecutive days prior to the onset of TBI, and for another 14 days thereafter. *C*. *butyricum* was found to significantly ameliorate neurological dysfunction, brain edema, neurodegeneration, intestinal barrier permeability and blood–brain barrier impairment in parallel with an increase in the glucagon-like peptide (GLP)-1 levels in the colon in relation to the control treatment.

In a mice weight-drop model of TBI induction, a single strain of *Lactobacillus acidophilus* at 2 × 1010 CFU/mL was given on the day of injury and for 1, 3, and 7 days thereafter. Probiotic treatment was found to significantly improve the neurological manifestation of the injury: intestinal barrier permeability, neuroinflammation, brain edema, neuronal loss, and sensorimotor deficits in relation to the placebo treatment group [72]. Furthermore, *L*. *acidophilus* demonstrated a decreased abundance of the family *Pseudomonadaceae* and an increase in *Prevotellaceae*, while controls exhibited a progressive, injury-severity-dependent increase in *Alpha*-*proteobacteria*, and specifically of the *Porphyromonadaceae* and *Pseudomonadaceae* families [47,50].

Recently, Cui et al. [73] randomized 40 male adult rats surgically subjected to a standardized brain trauma to receive either combination of a brain protein plus *Bifidobacterium lactis* or placebo. After 7 days of treatment, a decreased secondary inflammatory response and blood–brain barrier repair were prominent in *B*. *lactis* treated rats, in parallel with an increase in the immunoregulatory regulatory T cells (Tregs), in addition to a significant enhancement in the cerebral expression of ZO-1 and occludin proteins related to the strengthening of the blood–brain barrier and a decrease in the secondary inflammatory response of brain tissue.

Wild-type mice were subjected to controlled cortical impact injury and subsequently received daily, for the next 3 days, *Akkermansia muciniphila* [10 9 CFU] in 200 μL sterile phosphate-buffered saline. At the 7th post-injury day, *A*. *muciniphila*-treated mice presented with reduced intestinal mucosal injury and a better neurofunctional score in comparison to the control treated at days 3, 7, and 14 [74].

Based on the above, probiotic administration seems to alleviate TBI-induced gut dysbiosis in standardized animal models, mimicking different mechanisms of brain injury. Animal models, despite their advantages, still have limitations that need to be addressed. Human TBI is a complex disease, very difficult to be accurately reflected by animal models. Consequently, findings from experiments in animals cannot fully translate to humans. Thus, it is extremely important to further develop and increasingly use larger species with brains that are more anatomically and functionally closer to man. In addition, more research into the effect of age, sex and animal species or probiotic strain on the outcome of TBI is necessary. Larger as well as well-designed animal studies are required to extrapolate these preclinical findings to humans.

### 4.2. Human Studies

Several human clinical studies conducted to date have shown a reduction in unfavorable outcomes among TBI patients which received probiotics during hospitalization. These studies demonstrated that probiotic supplementation in patients with brain injury could effectively normalize the altered GM and decrease TBI-related complications such as lung, urinary tract and intracranial infections. There is also evidence that probiotics could prevent TBI-related GI symptoms such as diarrhea, vomiting, abdominal distension, constipation, stress ulcer and reflux. Finally, it was found that probiotic administration reduced time spent in the ICU and mechanical ventilation duration, as well as life-threatening conditions such as sepsis and multiple organ failure [75,76,77].

A randomized controlled trial (RCT) evaluated the effect of probiotics’ administration plus early enteral nutrition in 23 severe-TBI patients in the ICU. Eleven of them were randomly assigned to the probiotic group—240 mL of fermented milk with the probiotic strain *Lactobacillus johnsonii* plus 30 g of glutamine daily—and 12 received only a polymeric diet. The results demonstrated that the probiotic group had a significantly lower incidence of infections, shorter length of ICU stay and fewer days on mechanical ventilation than the control group [78].

In another single-blind RCT, 52 severe-TBI patients were randomized to receive seven sachets of a three-probiotics mixture composed of *Bifidobacterium longum* [0.5 × 108 cfu], *Lactobacillus bulgaricus* [0.5 × 107], and 0.5 × 107 *Streptococcus thermophilus* [0.5 × 107] or placebo. Probiotic-treated patients were found to have a lower incidence of nosocomial infections, shorter ICU stay and reduction in IL-4 and IL-10 levels [79]. Similar to previous studies, severe-TBI patients who received *Lactobacillus bulgaricus* plus enteral nutrition within the first 48 h of hospitalization experienced a shorter length of ICU stay and fewer days on mechanical ventilation, as well as lower nosocomial infection rates in relation to those on the placebo treatment [80].

Seventy-six severe-TBI patients were also randomized into probiotics—*Bifidobacterium longum*, *Lactobacillus bulgaricus*, and *Enterococcus faecalis*—or control. The probiotic-treated group demonstrated significantly reduced serum levels of IL-6, IL-10, and tumor necrosis factor TNF-α, along with reduced hospital stays and lower rates of respiratory infection. Moreover, GCS at 15 days was significantly lower in the probiotic group compared to the control group [81]. The same probiotic regime when administered in 68 severe-TBI patients led to a significant reduction in inflammatory response, immune function and TBI-related complications compared to a group of 68 similar patients treated with placebo [82].

A recent small-scale pilot RCT tested the efficacy of *Lactobacillus Reuteri* versus placebo in 31 U.S. war veterans with a history of mild TBI and (PTSD) symptoms. Probiotic supplementation for 8 weeks was associated with a significant decrease in serum CRP levels, suggesting that CRP may be a useful biomarker to monitor treatment effect in future studies, although the scope of this study did not include the detection of treatment-related changes in clinical PTSD symptoms [83].

In a more recent, phase IIa, randomized placebo-controlled trial, the same author discussed an experimental design to assess the effects of daily oral administration of *Lactobacillus rhamnosus* (1010 CFU) in 118 US military Veterans with TBI suffering from symptoms related to PTSD. Participants were equally but randomly assigned to either a probiotic or a placebo group, wherein each participant consumed one LGG or placebo capsule daily, for 8 ± 2 weeks. Given the chronic inflammation state among TBI patients and the anti-inflammatory effects of *Lactobacillus rhamnosus*, the primary outcome measure of this study is the plasma concentration of high-sensitivity CRP [84].

A meta-analysis of four studies (out of 8.781 articles identified) including 325 patients with severe TBI and post-traumatic stress disorder (PTSD) who received prebiotics/probiotics during hospitalization was carried out [85]. Two RCTs included participants who received probiotic treatment, which have previously been described in detail [78,79]. The retrospective cohort study by Painter and colleagues was not discussed here as they used an immune-enhancing nutrition formula containing prebiotics [86]. Finally, the fourth study by Gocan et al. [87], who examined the effects of a fermented soy formulation on veterans with PTSD suffering from depression and anxiety, was not included in this review. Although the findings suggested some promise about the beneficial effects of probiotics in TBI patients, the research in this area remains very limited with significant heterogeneity among the studies in terms of supplements administered, dosing strategies, timing, as well as the duration of administration. Given the widespread use of probiotics and their potential therapeutic effects, there is a need for larger prospective trials to explore the efficacy of specific probiotic supplements for patients with TBI.

In 2019, Yi et al. [88] conducted a meta-analysis of 18 RCTSs, which included 1016 patents between 2010 and 2016. The authors concluded that early enteral nutrition supplemented with probiotics in patients with severe head injury resulted in a significant reduction in infections, mortality rate and ICU length of stay. Similarly, another meta-analysis which included 39 studies found that probiotic supplementation in enteral nutrition resulted in a decreased risk of mortality, infection and gastrointestinal complications in patients suffering from a severe head injury [89]. These studies cover both Chinese and English language articles, resulting in a large number of articles included.

The published research on the use of probiotics in the acute care of TBI has mostly shown favorable findings with respect to decreased infections and improved outcomes. However, a recent systematic review and meta-analysis which reviewed seven RCTs indicated that the administration of probiotics in TBI patients had no significant effects on CRP, IL-6 and the length of staying in ICU. Thus, there was evidence of considerable data heterogeneity; a subsequent subgroup analysis was performed, which showed beneficial results. As a result, more carefully designed RCTs are needed to investigate the effect of probiotics on inflammatory biomarkers and ICU length of stay in TBI patients [90].

Overall, it appears that there is evidence of a positive effect from probiotic use on TBI outcomes. Probiotic treatment in the clinical context seems to be most beneficial in reducing infection, morbidity and mortality rates and decreasing inflammation. However, no studies exist examining the neurological or cognitive outcomes of probiotic supplementation on TBI patients. Additionally, probiotics are widely available for use in preclinical and clinical settings, they are relatively inexpensive, and have been generally demonstrated to be safe for human consumption. However, there is still a relative sparsity of human clinical studies on this subject, and a greater amount of research is needed to be able to more accurately determine the safety and efficacy of probiotic treatment among TBI patients before recommendations for clinical use. Furthermore, the vast majority of microorganisms colonizing the gut have remained unidentified until recently as they are mostly facultative anaerobe bacteria and, consequently, difficult to cultivate. With the development of human genome sequencing and techniques, newly beneficial bacterial strains have been isolated, cultured, and researched, defined as next-generation probiotics (NGPs) [91]. Among these, *Akkermansia muciniphila*, *Faecalibacterium prausnitzii*, *Eubacterium hallii Bacteroides uniformis*, *Bacteroides coprocola*, *Parabacteroides distasonis*, *Parabacteroides goldsteinii*, *Hafnia alvei*, *Odoribacter laneus* and *Christensenella minuta* have demonstrated efficacy in improving gut barrier integrity, exerting anti-inflammatory effects, modulating intestinal flora, and ameliorating intestinal dysbiosis in patients affected by neurodegenerative diseases [92]. Although there are many studies confirming the beneficial effects of traditional probiotics on TBI, repeated preclinical experiments on mice, clinical human trials and complex screening processes are needed to go beyond investigating the exact benefits of NGPs on TBI-patients.

## 5. Conclusions

As TBI constitutes a major public health issue worldwide, more preventive and therapeutic approaches against TBI and its related complications are urgently needed. Growing clinical evidence reveals that targeting the GMΒA is of great importance in the treatment of TBI patients and there is increasing evidence about the beneficial properties of probiotics among these patients. In this review of the literature, we summarize preclinical and clinical studies that demonstrate the potential importance of gut dysbiosis in the pathogenesis and progression of TBI complications. We also highlighted the significance of modulating the composition of gut microbiome by probiotics as an important and promising therapeutic strategy to promote gut eubiosis and immune function and modulate inflammatory responses.

However, there are still gaps in the literature that need to be addressed. To confirm the positive effects of probiotics on TBI, and to determine the optimal strain and dose with clinical efficacy, as well as timing and duration of administration, more well-designed randomized controlled clinical trials should be conducted. Further research on new-generation probiotics regarding their effects on TBI patients should be verified in animal experiments and human trials, which would lead to the development of novel therapeutic approaches to improve the intestinal barrier, alleviate symptoms, and allow patients suffering from TBI to recover quickly from disease.
